# Evaluation of vaccination coverage and the knowledge of parturient admitted for labor in a public tertiary maternity hospital in western São Paulo, Brazil

**DOI:** 10.3389/fpubh.2025.1542321

**Published:** 2025-10-31

**Authors:** Luís Antônio Gilberti Panucci, Luiza Sant’Anna Pinheiro, João Pedro Teixeira Roque, Edilson Ferreira Flores, Rogério Giuffrida, Luiz Euribel Prestes-Carneiro

**Affiliations:** ^1^Hospital Estadual de Presidente Prudente, Presidente Prudente, Brazil; ^2^Faculty of Medicine, University of Western São Paulo, Presidente Prudente, Brazil; ^3^Department of Statistics, Faculty of Science and Technology, São Paulo State University, Presidente Prudente, Brazil; ^4^Postgraduate Program in Health Sciences, University of Western São Paulo, Presidente Prudente, Brazil

**Keywords:** prenatal card, Brazilian National Immunization Program, vaccination coverage, pregnancy, misinformation pregnant card, vaccines, vaccine knowledge, maternal education

## Abstract

**Introduction:**

Vaccination coverage among pregnant women in Brazil remains poorly documented, particularly in low-resource settings. This study aimed to (1) assess the completeness of vaccine records on prenatal cards and (2) evaluate pregnant women’s knowledge of vaccines recommended by the Brazilian National Immunization Program (NIP).

**Methods:**

: A cross-sectional study was conducted at the Hospital Estadual de Presidente Prudente (HEPP), a public secondary hospital in São Paulo, Brazil, between August 2022 and April 2023. The study population comprised 1,130 women admitted for delivery, of whom 541 (47.9%) had prenatal cards available for review. Postpartum, data from the prenatal cards were extracted, and participants completed a structured questionnaire to assess their vaccine knowledge. Sociodemographic data were obtained from electronic medical records. Univariate analyses were performed using Pearson’s Chi-Squared or Fisher’s Exact Test. Only 11.2% of the reviewed prenatal cards documented complete vaccination with all four NIP-recommended vaccines, while 31% contained no vaccine records at all. The tetanus, diphtheria, and acellular pertussis (Tdap) vaccine had the highest documented coverage (61%). The mean age of participants was 27.1 ± 0.3 years. Although most participants (91.1%) believed they had been vaccinated during pregnancy, only 61.5% could specify which vaccines they had received. No significant association was found between sociodemographic factors and the completeness of vaccine documentation. The low level of vaccination documentation and the critical knowledge gaps identified in this study highlight deficiencies in the quality of prenatal care within Brazil’s Unified Health System (SUS) in the Western region of São Paulo state. Addressing these issues requires concerted efforts to improve healthcare provider training, strengthen public health education, and standardize documentation practices.

## 1 Introduction

Vaccination during pregnancy is essential for protecting both mothers and newborns from infectious diseases (1, 2). Newborns depend on maternal antibodies, transferred transplacentally and through breastfeeding, to acquire critical immunity during early infancy (3). In Brazil, the National Immunization Program (NIP) oversees population-wide vaccination efforts, prioritizing pregnant women and children (4, 5). The NIP recommends four key vaccines during pregnancy: tetanus, diphtheria, and acellular pertussis (Tdap), hepatitis B, influenza, and COVID-19 (6).

Since 1988, the prenatal card has served as a mandatory health record in Brazil’s public and private healthcare systems, documenting obstetric care, test results, and vaccinations (6, 7). This tool is critical for recording essential health information, including vaccination status, which guides healthcare providers during childbirth (8). Despite its importance, studies using prenatal card data have revealed systemic issues, such as incomplete documentation and inconsistent record-keeping within the Unified Health System (SUS) (4, 9). Although the Brazilian Ministry of Health updated the prenatal card in 2022 to improve usability (7), challenges persist in adequately training healthcare teams to maintain accurate records (4, 9). The COVID-19 pandemic further exacerbated these issues, as disruptions in healthcare services during 2020–2021 led to declines in vaccination coverage and increased risks of preventable diseases (10).

Globally, education is a key determinant of health outcomes, influencing indicators such as infant mortality, vaccination rates, and life expectancy (11). Even in high-income countries, lower levels of educational attainment are associated with reduced health literacy and limited healthcare access (12). Nevertheless, the impact of maternal education on vaccination adherence remains underexplored, particularly in low-resource settings (13, 14).

In Brazil, despite NIP efforts to achieve 90% coverage among pregnant women, national Tdap coverage reached only 87.9% by 2024 (15, 16). Clinical observations by the authors at the Hospital Estadual de Presidente Prudente (HEPP) revealed a notable decline in the registration of vaccinations on prenatal cards during the COVID-19 pandemic. This led to the hypothesis that, in the western region of São Paulo state, primary care professionals may be insufficiently trained to record vaccination data accurately, resulting in pregnant women receiving inadequate information about the importance of immunization. Therefore, this study aimed to (1) assess the completeness of vaccine records on prenatal cards and (2) evaluate pregnant women’s knowledge of vaccines recommended by the NIP.

## 2 Methodology

### 2.1 Study area and design

This cross-sectional study was conducted in the western region of São Paulo, one of the state’s 18 Regional Health Care Networks (RHCN) (17). This region comprises 45 municipalities with a total population of approximately 753,000 (18). The study was set at the Hospital Estadual de Presidente Prudente (HEPP), a tertiary public hospital that serves as a regional referral center for high-risk pregnancies within Brazil’s Unified Health System (SUS).

### 2.2 Data collection from pre-natal cards and structured questionnaire

The study was conducted between August 1, 2022, and April 30, 2023. Women admitted for delivery at HEPP were considered for inclusion. Upon admission, sociodemographic data—including municipality of residence, educational level, marital status, and occupation—were obtained from the hospital’s electronic records.

During the immediate postpartum period, an obstetrician invited eligible women to participate. For those who provided informed consent, two data collection procedures were performed. First, the pages of the participant’s prenatal card containing identification and vaccination records were photographed, and the data were transcribed into a digital spreadsheet. Second, the same obstetrician administered a structured questionnaire adapted from validated instruments (19–21). The questionnaire included items assessing participants’ awareness of maternal vaccines, the perceived adequacy of the information they had received, and their understanding of maternal antibody transfer.

### 2.3 Association of epidemiological variables and dTpa, HBV, influenza and COVID-19

**Figure 1:**
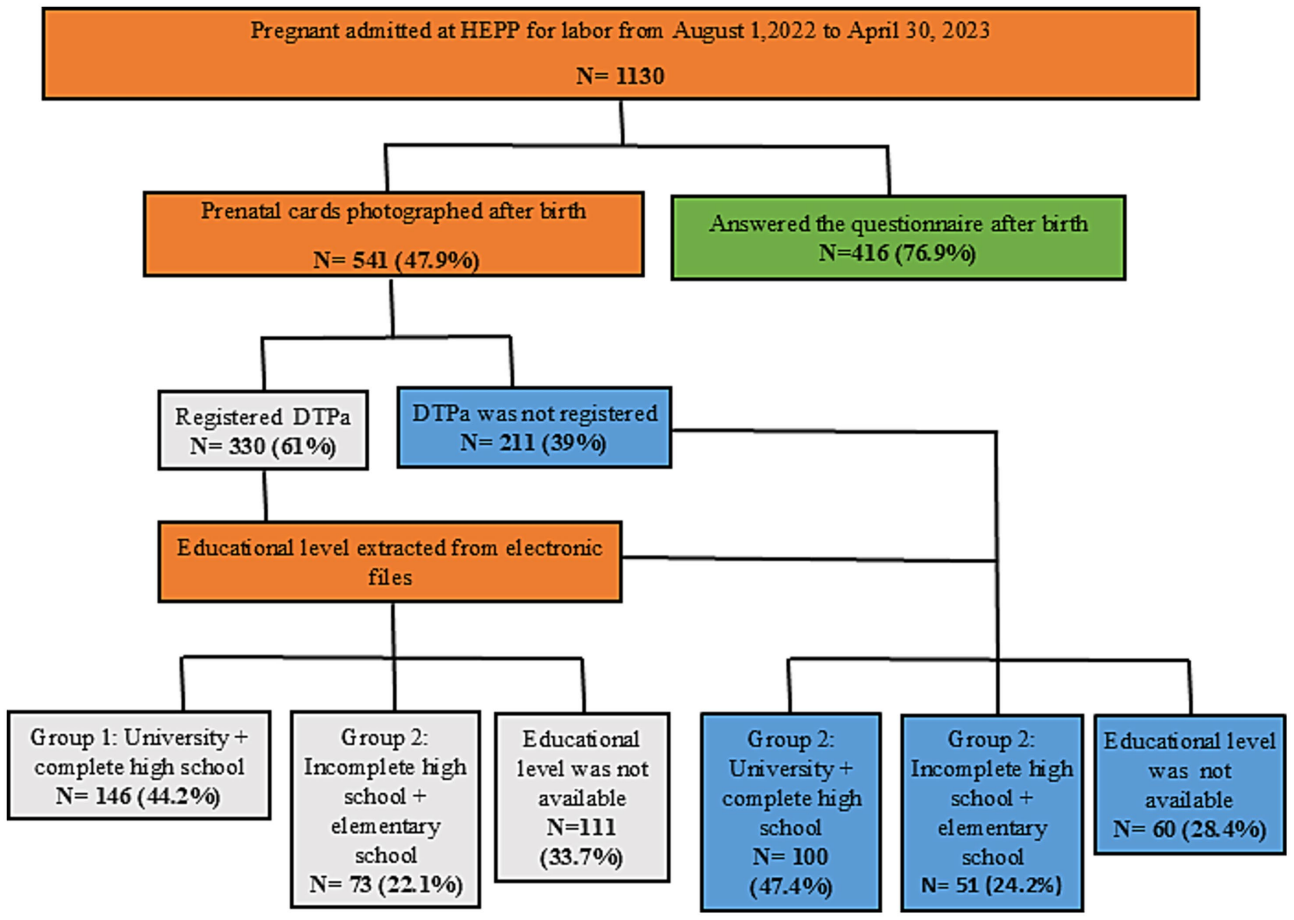
Flow chart illustrating the study design for pregnant women admitted for labor at HEPP. Colors indicate the different maternal education groups: Group 1 (white) represents higher education, defined as having a university degree (complete or incomplete) or having completed high school; Group 2 (blue) represents lower education, defined as having incomplete high school or only elementary education. dTpa, acellular diphtheria-tetanus-pertussis.

The primary association examined was between the registration of the tetanus, diphtheria, and acellular pertussis (Tdap) vaccine on prenatal cards and maternal education level. The Tdap vaccine is a key indicator used by the Brazilian Ministry of Health to monitor vaccination in vulnerable groups, including pregnant women (16). Maternal education was categorized into two groups based on data from electronic medical records: higher education (completed high school or a complete/incomplete university degree), depicted in white in Figure 1; and lower education (incomplete high school or only elementary education), depicted in blue in Figure 1.

The secondary associations examined were between the registration of the other NIP-recommended vaccines (hepatitis B, influenza, and COVID-19) and other epidemiological variables (age, race, marital status, and occupation). Data for all variables were extracted from electronic records. Records with incomplete or inconsistent data were excluded from the corresponding analyses.

### 2.4 Statistical analysis

All statistical analyses were performed using R software (R Core Team, 2024). Continuous variables, such as age, were reported as mean ± SEM, while categorical variables were presented as frequencies and percentages (%). Statistical significance for all tests was defined as a *p*-value < 0.05.

The association between maternal education and Tdap registration was assessed using the chi-square test with Yates’ correction, and odds ratios (OR) with 95% confidence intervals (CI) were calculated. For the secondary analyses, associations between other vaccine registrations and epidemiological variables were assessed using Pearson’s Chi-Squared Test or Fisher’s Exact Test. Corresponding odds ratios and 95% confidence intervals were also calculated for these factors.

## 3 Results

### 3.1 Profile of vaccination coverage documented on prenatal cards

During the study period, 1,130 women were admitted for delivery at HEPP. Among these, prenatal cards were available for review for 541 women (47.9%), who constituted the study sample (Figure 1).

**Figure 2:**
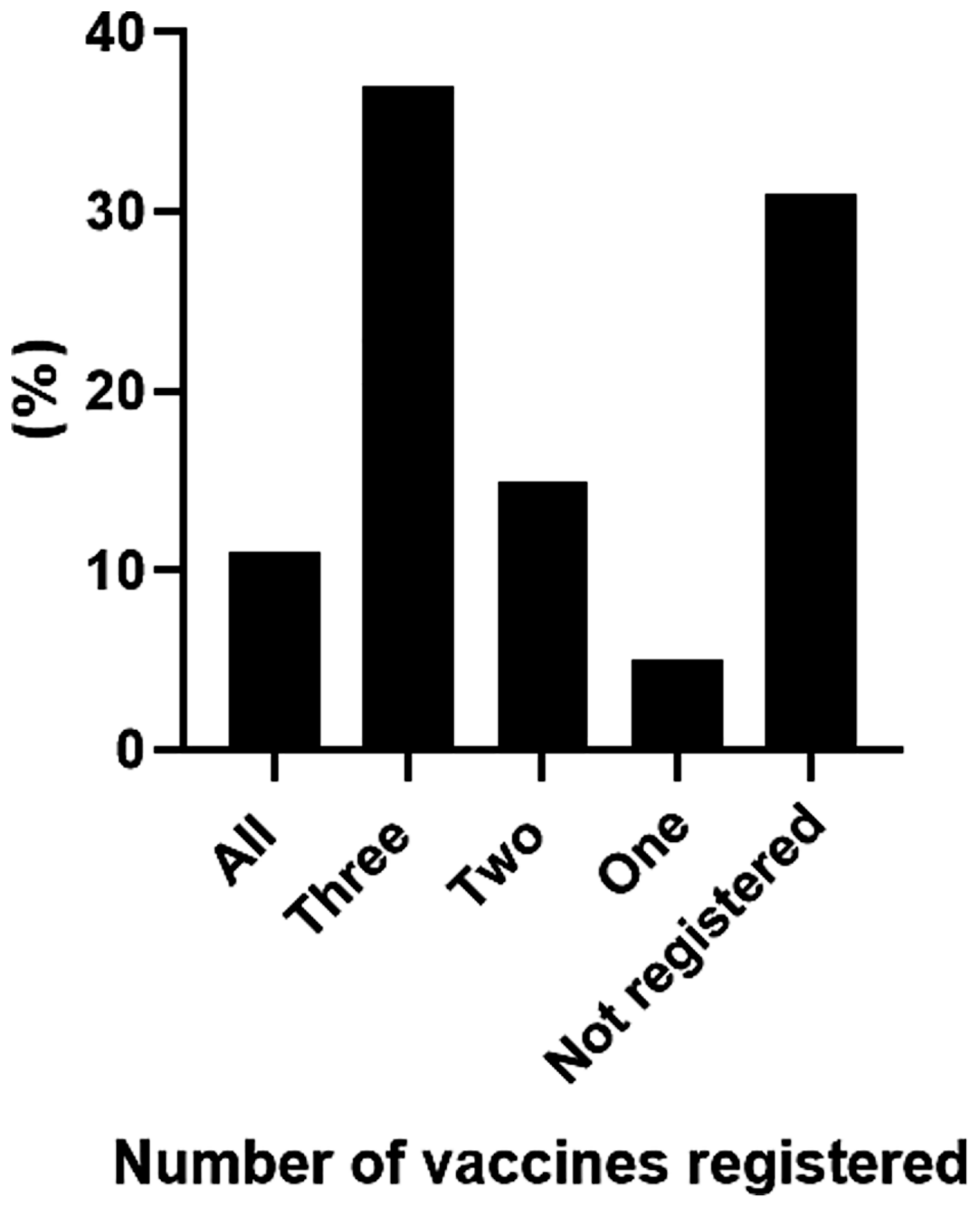
Percentage of vaccines registered in the prenatal cards. Prenatal cards of pregnant women admitted for labor at HEPP. The columns represent the number of vaccines registered, with the final column indicating cases where no vaccination was recorded, from August 2022 to April 2023.

Of the reviewed cards, complete vaccination (documentation of all four NIP-recommended vaccines) was recorded on only 61 cards (11.2%). A partial record of three vaccines was found on 203 cards (37.5%), two vaccines on 86 cards (15.8%), and a single vaccine on 23 cards (4.2%). Notably, 168 prenatal cards (31.0%) contained no vaccination records whatsoever (Figure 2).

**Figure 3:**
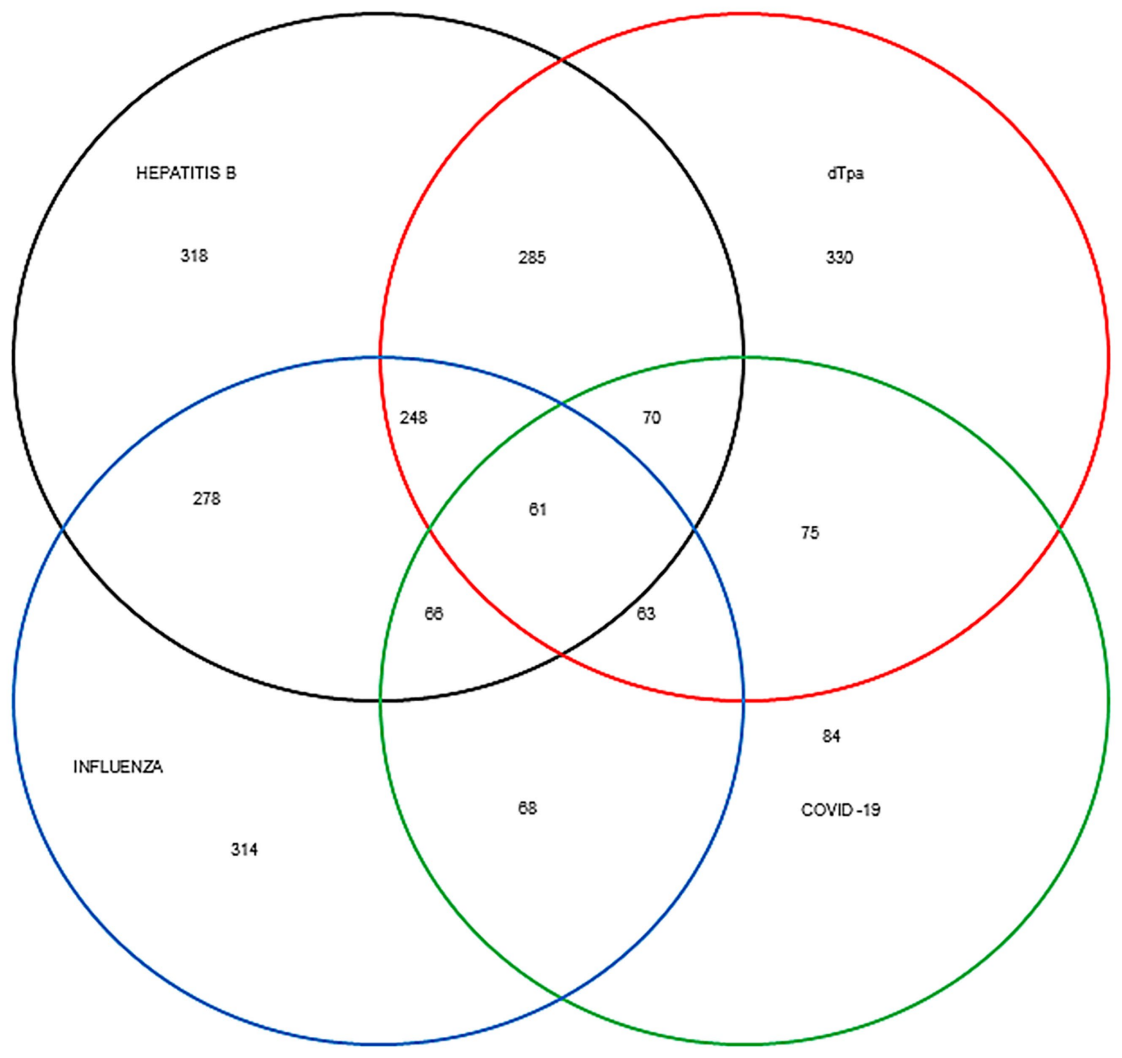
Venn diagram providing a schematic representation of registered vaccines. The proportion of pregnant women who received 2, 3, or 4 vaccines, as registered on prenatal cards from August 2022 to April 2023. dTpa, acellular diphtheria-tetanus-pertussis.

The tetanus, diphtheria, and acellular pertussis (Tdap) vaccine had the highest documented coverage, appearing on 330 cards (61.0%), whereas the COVID-19 vaccine had the lowest, with only 84 records (15.5%) (Figure 3). The most common two-vaccine combination was Tdap and hepatitis B (*n* = 285). Among women with three vaccines recorded, the most frequent combination was Tdap, hepatitis B, and influenza (*n* = 248).

**Figure 4:**
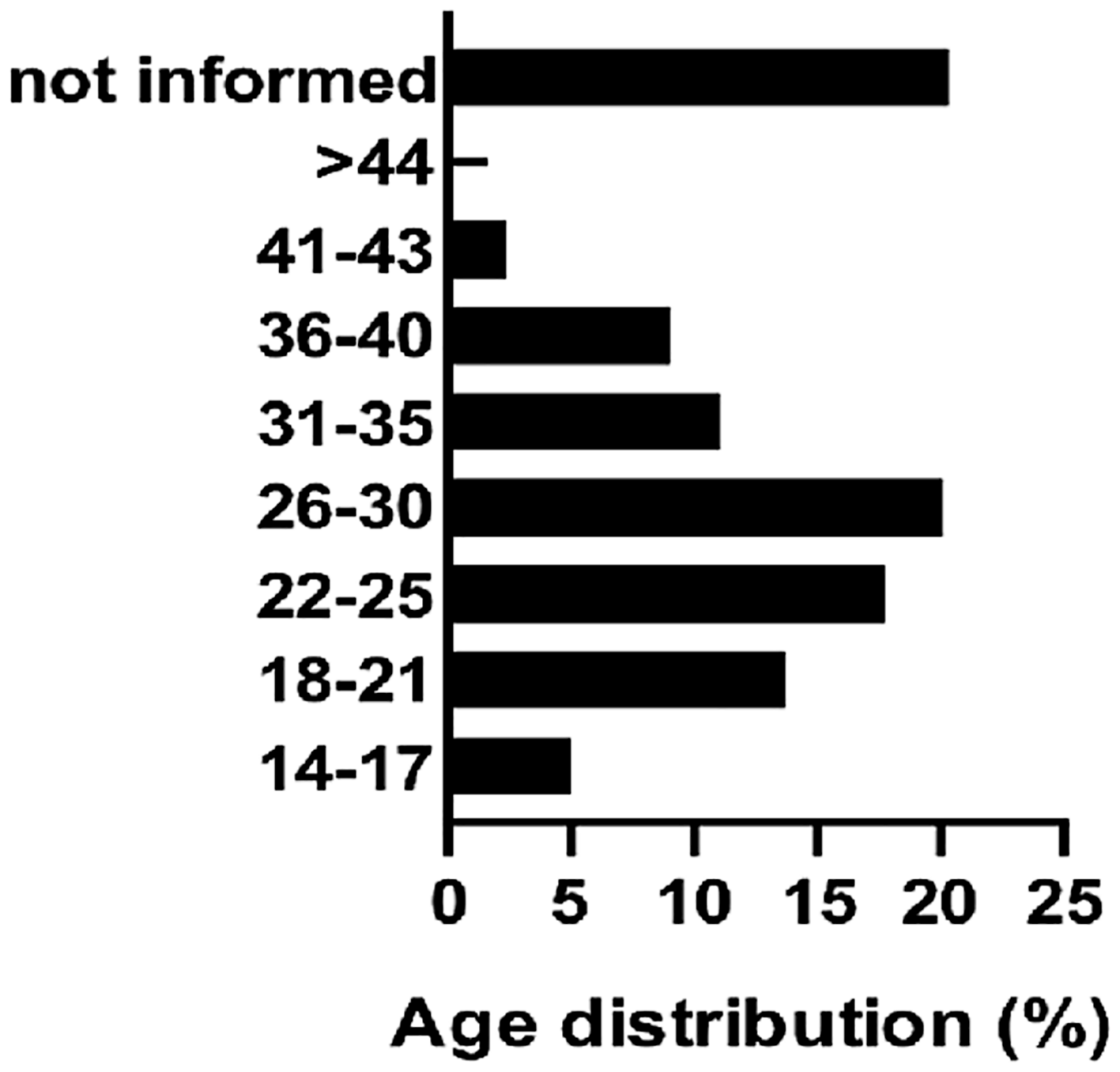
Percent of age distribution of pregnant women. Age was registered in the Prenatal Cards of Women Admitted for Labor at HEPP (August 2022–April 2023). Columns represent different age groups.

The mean age of the participants for whom data was available was 27.1 ± 0.3 years (95% CI, 26.4–27.7, range, 14–50 years, n = 431). The largest proportion of participants (20.8%) belonged to the 26–30-year age group, while adolescents (14–17 years) constituted 4.9% of the sample (Figure 4).

### 3.2 Knowledge regarding NIP-recommended vaccines during pregnancy

**Figure 5:**
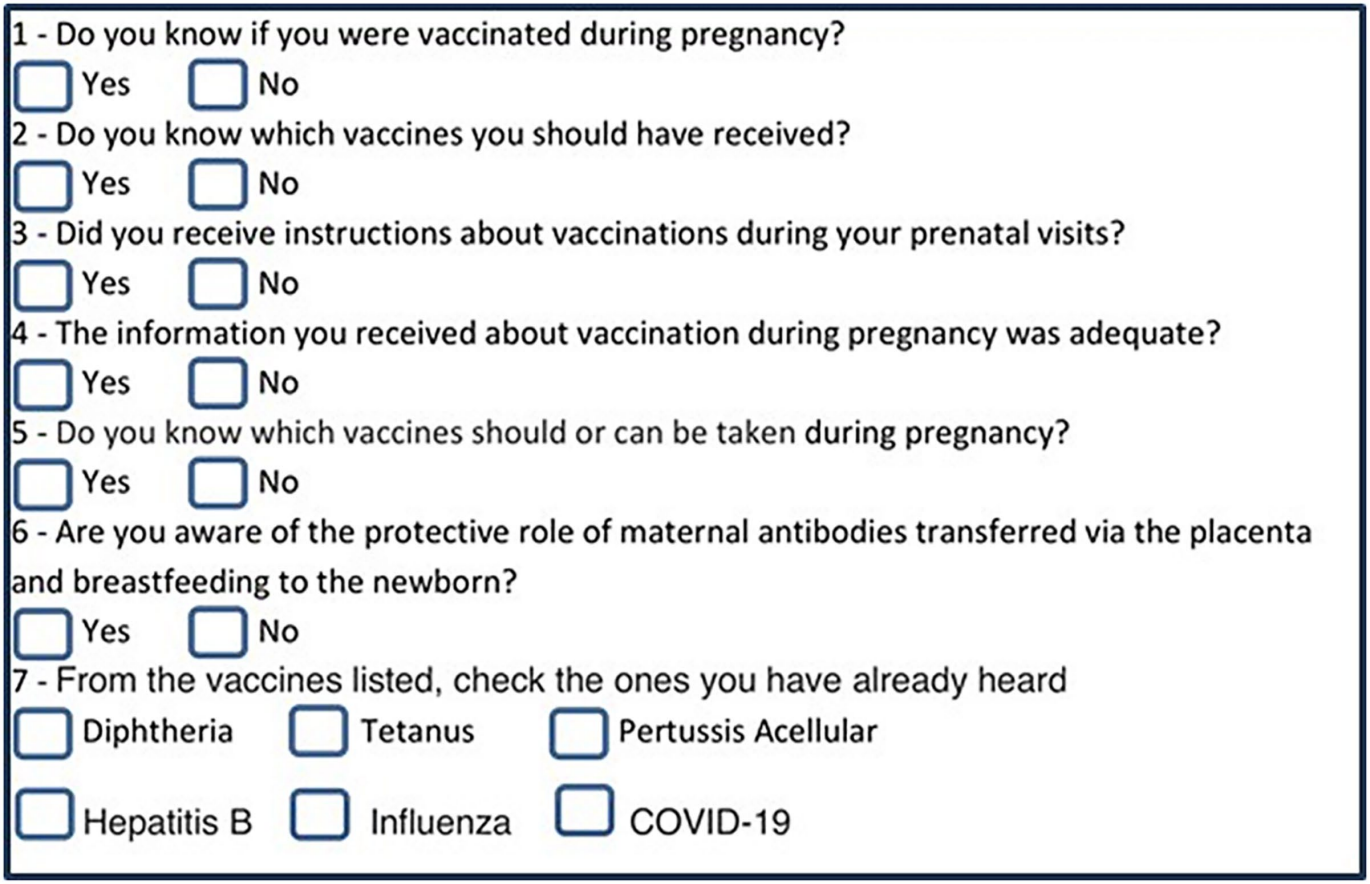
Survey questions assessing the knowledge of vaccination. The structured questionnaire was applied among patients admitted for labor at HEPP (August 2022–April 2023).

Of the 541 women in the initial sample, 416 (76.9%) completed the structured questionnaire (Figure 1). The survey’s structure and the knowledge domains it assessed are illustrated in Figure 5.

The findings on vaccination knowledge are presented in Table 1. While a high proportion of participants (91.1%) knew they had been vaccinated during their pregnancy, only 61.5% could specify which vaccines they had received. Most women (80.8%) reported receiving some instruction about vaccination from healthcare providers; however, only 42.5% considered this information adequate. Furthermore, a substantial number of participants (74.0%) were unaware of the specific vaccines they were eligible to receive during pregnancy. Regarding the protective role of maternal antibodies, 62.0% of respondents indicated awareness.

**Table 1:** Data regarding vaccination knowledge of 416 pregnant women admitted for labor at HEPP (August 2022–April 2023).

Data	Yes no (%)	No no (%)
1 - Do you know if you were vaccinated during pregnancy?	379 (91.1)	37 (8.9)
2 - Do you know which vaccines you have received?	256 (61.5)	160 (38.5)
3 - Did you receive instructions about vaccinations?	336 (80.8)	80 (19.2)
4 - Was the information you received adequate?	177 (42.5)	239 (57.5)
5 - Do you know which vaccines should or can be taken?	108 (26)	308 (74)
6 - Are you aware of the protective role of maternal antibodies?	258 (62)	158 (38)
7 - From the vaccines listed, check the ones you have already heard		
Diphtheria	100 (24%)	
Tetanus	392 (94.2)	
Acellular pertussis	174 (41.8)	
Hepatitis B	381 (91.6)	
Influenza	343 (82.5)	
COVID-19	389 (93.5)	

In terms of name recognition for specific vaccine-preventable diseases, participants were most familiar with tetanus (94.2%), followed by COVID-19 (93.5%) and hepatitis B (91.6%). Diphtheria was the least recognized disease (Table 1).

### 3.3 Association of epidemiological variables and dTpa, HBV, influenza and COVID-19 vaccination

The primary analysis focused on the association between maternal education and Tdap vaccine registration among the 541 participants with available prenatal cards. Overall, Tdap vaccination was recorded on 61.0% of the cards and was absent from 39.0% (Figure 1). Among participants with a Tdap record, 44.2% had a higher educational level and 22.1% had a lower educational level; data on education was missing for the remaining 33.7%. Among those without a Tdap record, 47.4% had a higher educational level and 24.2% had a lower educational level, with data missing for 28.4%.

The proportion of Tdap registration was comparable between educational levels: 59.3% of the higher education group had a vaccine record, versus 58.9% of the lower education group. A chi-square test confirmed that there was no statistically significant association between maternal education and Tdap registration (χ^2^ = 0.008, *p* = 0.93). Furthermore, logistic regression analysis confirmed the absence of a significant link (OR = 1.03; 95% CI: 0.64–1.67).

Finally, a univariate analysis found no significant association between the registration of any of the four NIP-recommended vaccines (Tdap, hepatitis B, influenza, and COVID-19) and other epidemiological variables, including maternal age, race, marital status, and occupation (Table 2).

**Table 2:** Association of one or more vaccines registered in prenatal cards with epidemiological characteristics of pregnant women admitted for labor (*N* = 279) in HEPP, univariate analysis.

Variables	One or more vaccines		OR (95% CI)	*p*-value
	yes (%)	no (%)		
	196 (70.3)	83 (29.7)		
Age (years)				0.674
14–21	48 (24.5)	23 (27.7)	Ref.	
22–30	95 (48.5)	36 (43.4)	1.26 (0.67–2.37)	
31–40	47 (24.0)	23 (27.7)	0.98 (0.48–1.99)	
> 40 years	6 (3.06)	1 (1.20)	2.57 (0.39–69.3)	
Race				0.206
White	91 (46.4)	31 (37.3)	Ref.	
No white	105 (53.6)	52 (62.7)	0.69 (0.40–1.16)	
Civil status				0.454
Married or not	136 (69.4)	62 (74.7)	Ref.	
Divorced or single	60 (30.6)	21 (25.3)	1.30 (0.73–2.36)	
Occupation housewife				0.902
No	93 (47.4)	38 (45.8)	Ref.	
Yes	103 (52.6)	45 (54.2)	0.94 (0.56–1.57)	

OR, Odds ratio; CI, Coefficient interval.

## 4 Discussion

This study reveals critical gaps in both vaccination documentation and knowledge among pregnant women in the western region of São Paulo. A key finding was that only 11.2% of prenatal cards documented all four NIP-recommended vaccines, while an alarming 31% contained no vaccination records at all. Furthermore, while most participants knew they had been vaccinated, just over 60% could specify which vaccines they had received, and less than half considered the information provided by healthcare professionals to be adequate. Together, these findings underscore systemic challenges in the quality of prenatal care and the accuracy of health records within the SUS.

The low rate of complete vaccine registration (11.2%) observed in this study is a significant concern. This issue was first noted clinically by the authors at HEPP during the COVID-19 pandemic and appears to have persisted. Several factors may explain these poor documentation rates. On the provider and system side, potential factors include a lack of awareness among healthcare professionals regarding current vaccine recommendations and failures at primary care centers to verify and transcribe all necessary information onto the prenatal card (22). The specific context of the western region of São Paulo, which comprises many small municipalities, may also play a role, as healthcare professionals in these areas may not be sufficiently prepared to provide comprehensive counseling on maternal immunization (17).

On the patient side, additional factors may contribute, including concerns about vaccine safety for the mother or newborn, apprehension about side effects, doubts regarding efficacy, and the proliferation of misinformation (22). Fears of contracting COVID-19 at healthcare facilities, especially during 2020–2021, likely also contributed to decreased engagement with routine health services, including vaccination (10).

In Brazil, the health-seeking behaviors of pregnant women are aimed at safeguarding their own health and that of their babies. These behaviors, which include attending prenatal care, seeking help for complications, and preparing for delivery, are shaped by determinants such as education, socioeconomic status, cultural beliefs, and knowledge of pregnancy’s danger signs. However, pregnant women face significant challenges, including poor access to care, prevalent misinformation, and social inequalities, even though prenatal care is a public health priority (5, 8, 9).

Trust in the public healthcare system (SUS) varies greatly across the country’s diverse regions. This trust is often influenced by socioeconomic inequality, educational levels, a lack of effective guidance from providers, and potential discrimination. For instance, in regions like the Northeast and the Amazon, which are often characterized by high poverty rates, low educational attainment, and significant geographical barriers to healthcare, trust in the system can be particularly low. Moreover, even in the peripheries of large cities in more developed states, pregnant women’s confidence in the public health system is often compromised (4, 5, 8, 16).

The low completeness of vaccine registration observed in our study is consistent with findings from other regions in Brazil. For example, a study in Botucatu (São Paulo) reported that 68.4% of pregnant women received recommended vaccines a rate significantly higher than the complete coverage found in our study, yet still below NIP targets (22). Similarly, research in Greater Vitória (Espírito Santo) reported 59.3% Tdap vaccine coverage, highlighting documentation inconsistencies across public health services (23). Furthermore, a national study documented a sharp decrease in Tdap coverage for pregnant women to 45.4% in 2020 from 63.2% in 2019, far from the national target of 95% (4, 16). While Latin American influenza vaccination rates average around 59% (24), even high-income countries like the U. S. and Italy report persistent gaps in maternal vaccine uptake despite robust healthcare infrastructures (14, 25). Collectively, these trends point to systemic barriers including misinformation, insufficient provider training, and logistical challenges That impede vaccination efforts worldwide (22–25).

Our analysis of vaccine combinations, illustrated in a Venn diagram, revealed specific patterns in partial vaccination. The most common two-vaccine combination was Tdap and hepatitis B (HBV), while the most frequent three-vaccine combination was Tdap, HBV, and influenza. These results align with our questionnaire findings, in which Tdap, hepatitis B, and influenza were, in that order, the most commonly recognized vaccines. In contrast, a study in Italy reported that the most frequently recalled combination during pregnancy was Tdap and COVID-19 (26). This contrast highlights the low COVID-19 vaccination coverage observed in our study and across Brazil. Although COVID-19 vaccination during pregnancy is safe and effective in reducing complications for both the mother and newborn, its uptake remains below targets worldwide (27). For instance, a large cohort study in Rio de Janeiro reported that only 53.0% of pregnant women had received at least one dose of a COVID-19 vaccine (28).

The mean age of the participants was 27.1 years, with a range of 14 to 50 years. The largest age group was 26–30 years (20.8%); however, a substantial proportion (18.6%) of participants were adolescents and young adults (aged 14–21 years) (Figure 5). This high prevalence of young mothers reflects a demographic reality frequently observed in the maternity wards at HEPP and raises important public health considerations regarding maternal and child well-being.

Several factors may contribute to the high rate of adolescent and young adult pregnancy in this population. One significant factor is the intergenerational cycle of early childbearing, wherein daughters of teenage mothers have a higher risk of becoming teenage mothers themselves. This cycle is often linked to poverty, lower educational attainment, and limited access to healthcare and other opportunities (14, 24, 29). The study population, drawn entirely from the SUS, is largely characterized by these socioeconomic vulnerabilities.

Although adolescent pregnancy rates have been decreasing, they remain high in Brazil compared to global averages (29). National studies confirm this trend; for example, data from the 2013 National Health Survey showed that a large proportion of first pregnancies in Brazil occur during adolescence, particularly in regions with poorer socioeconomic conditions (9). Similarly, a large study of SUS users found that women aged 12–19 years accounted for 21.4% of pregnant and postpartum patients analyzed (8). This context is particularly relevant to the western region of São Paulo, which is one of the state’s most socioeconomically challenged areas, with several municipalities exhibiting low Human Development Index scores (30). While our study did not assess socioeconomic status directly, this underlying vulnerability is a recognized characteristic of pregnant women served by the SUS (31).

Knowledge gaps regarding vaccination persist among pregnant women worldwide (19, 20, 24, 25). In our study, participants most frequently recognized tetanus and COVID-19 by name; in contrast, the importance of protection against diseases like acellular pertussis and diphtheria was less understood. Only 57.5% of respondents felt sufficiently informed by healthcare professionals about maternal immunization, and a mere 38% were aware of the protective role of transplacental antibody transfer.

Defining the precise reasons for these knowledge gaps among pregnant women in western São Paulo is challenging. We suggest that while healthcare providers are seen as a reliable source of information, their recommendations alone may be insufficient. Systemic issues, such as high turnover among healthcare professionals within the SUS, can lead to disruptions in the continuity of prenatal care. Other factors, including low socioeconomic status, lower maternal education, and delayed initiation of prenatal care, also likely contribute to the observed knowledge gaps in our region.

Global evidence underscores the critical role of healthcare providers in enhancing vaccine uptake during pregnancy (19, 20, 24, 25). For example, a study in France reported that adherence to vaccination among pregnant women was positively associated with awareness of maternal vaccines, confidence in vaccine effectiveness, and receiving recommendations from healthcare professionals (20). Similarly, a U.S.-based study demonstrated that clear recommendations from providers, coupled with educational materials, significantly improved acceptance rates for both influenza and Tdap vaccines (19). In several Latin American countries, including Brazil, pregnant women exhibited limited knowledge regarding the role of maternal antibodies and the specific vaccines recommended during pregnancy (32). Collectively, these findings underline the importance of training healthcare professionals to deliver accurate, timely, and personalized vaccination counseling.

The Tdap vaccine, which protects against tetanus, diphtheria, and pertussis (whooping cough), is mandatory for all pregnant women in Brazil (6, 22). Given that most women admitted for delivery at HEPP experience high social vulnerability, we investigated whether higher maternal education was associated with a greater likelihood of having Tdap vaccination recorded on their prenatal cards. Our study found no significant association, challenging the common assumption that higher education invariably leads to better health-seeking behaviors.

A possible explanation for this result is the relative socioeconomic homogeneity of the study population. This homogeneity was reflected not only in the lack of association between education and Tdap registration but also in the absence of significant associations between other epidemiological variables (age, race, marital status, occupation) and the registration of other recommended vaccines. Our findings are in line with other Brazilian studies. Research in a São Paulo state city found no association between completing the postpartum vaccination schedule (Tdap and hepatitis B) and maternal education, age, race, or marital status (22). Similarly, a study in Belo Horizonte (Minas Gerais) found no link between tetanus vaccine uptake and maternal education (33).

In contrast, other studies have found an association. In Italy, higher educational levels were significantly associated with vaccination during pregnancy (14), and interventions in Latin America that included a provider education component positively impacted maternal vaccine uptake (34). These conflicting findings likely reflect the descriptive nature of many of these studies, which are subject to various confounding variables and biases that can differ by location and population.

Through Brazil’s Unified Health System (SUS), the country has achieved high prenatal care coverage, exceeding 97%, with most women receiving care in primary health units. In the western region of São Paulo (DRS-XI), SUS-affiliated prenatal healthcare is available in all municipalities, ensuring access to monitoring, testing, vaccination, and linkage to a maternity hospital for delivery (8, 15, 16, 23). However, high accessibility does not always translate to high vaccine uptake. In Brazil and elsewhere, individual perceptions of vaccine safety strongly influence pregnant women’s decisions, with fears about side effects or negative beliefs often leading to hesitancy (25, 26, 34).

This study has several strengths that underscore the reliability and value of its findings. First, it benefits from a large and representative sample, analyzing 47.9% of the 1,130 women admitted for delivery at HEPP during the study period, a major referral center for 45 municipalities. Second, the study has a comprehensive design, assessing three different aspects: the documentation of vaccines on prenatal cards, the association between epidemiological variables and vaccine registration, and the participants’ own knowledge of vaccination. Third, the data collection was rigorous and standardized, conducted by a single researcher to ensure consistency. Finally, the study has direct practical applications, providing valuable insights for developing regions facing similar challenges with vaccine uptake and documentation. The findings contribute significantly to the scientific literature and have the potential to be replicated at state or national levels.

This study has several limitations that should be noted. (i) The study was conducted exclusively in a public hospital (SUS). This may limit the generalizability of our findings, as pregnant women served by private healthcare providers who may exhibit higher vaccination coverage and more consistent documentation were not included. (ii) Our analysis was restricted to the standardized prenatal card issued by the Brazilian Ministry of Health (MS) to ensure data consistency. It is known that these standard cards are not always used in primary care centers; vaccination data are sometimes recorded on different documents or formats, which could have led to an underestimation of the true documentation rate. (iii) A significant portion of women did not have their prenatal cards available at the time of admission to HEPP, which restricted the sample size. This lack of documentation is a limitation in itself, as in clinical practice, the absence of a record is often interpreted as the absence of the procedure. Only 69.0% of patients had the vaccines registered in the Pregnancy Card at the time of admission in the HEPP limiting the conclusion of the scope of the study showing the poor quality of data entry by healthcare professionals, and this underreporting constitutes a limitation, as the lack of records presupposes that the procedures were not performed. (iv) The study is subject to the inherent biases of observational and retrospective designs. The questionnaire data, in particular, may be subject to recall bias, as participants’ memories of information received during pregnancy can be imperfect. Furthermore, the analysis of card records is subject to information bias due to reliance on potentially inaccurate or incomplete historical data. Finally, the observed associations may be influenced by unmeasured confounding variables.

## 5 Conclusion

Despite significant progress in expanding access to prenatal care in Brazil, this study reveals that the documentation of maternal vaccinations on prenatal cards remains critically low in the western region of São Paulo. This finding, coupled with the identification of significant knowledge gaps among pregnant women, raises serious concerns about the quality of prenatal counseling and the accuracy of health records within public health services. Our results show that even when women are vaccinated, they often lack adequate information about the specific immunizations they have received. Furthermore, this study challenges the common assumption that higher education invariably leads to better health practices, as we found no significant association between maternal education and Tdap vaccination registration in this population.

The challenges identified here—including low documentation rates, prevalent knowledge gaps, and the complex influence of socioeconomic factors—are not unique to this region. These findings likely reflect a broader national trend and may be applicable to other Latin American countries facing similar issues of suboptimal vaccination rates and widespread misinformation. Therefore, addressing these deficiencies requires a multi-pronged approach: concerted efforts must be made to improve the training of healthcare providers, strengthen public health education campaigns to empower pregnant women with clear information, and standardize documentation practices to ensure every vaccine counts.

## Data Availability

The raw data supporting the conclusions of this article will be made available by the authors, without undue reservation, upon request.

## References

[1] Sato APS Boing AC Almeida RLF Xavier MO Moreira RS Martinez EZ. Vacinação do sarampo no Brasil: onde estivemos e para onde vamos? Ciênc Saúde Colet. (2023) 28:351–62. doi: 10.1590/1413-81232023282.19172022 36651391

[2] Beeler JA Lambach P Fulton TR Narayanan D Ortiz JR Omer SB. A systematic review of ethical issues in vaccine studies involving pregnant women. Hum Vaccin Immunother. (2016) 12:1952–9. doi: 10.1080/21645515.2016.1186312, PMID: 27246403 PMC4994733

[3] Chaudhari T. Vaccinations in the newborn. Best Pract Res Clin Obstet Gynaecol. (2021) 76:66–82. doi: 10.1016/j.bpobgyn.2020.09.004, PMID: 33129673

[4] Mello LR Marano D Moreira MEL Domingues RMSM Costa ACC Dias MAB. Avaliação da completitude de preenchimento do cartão da gestante do Ministério da Saúde: estudo seccional, de âmbito nacional. Ciênc Saúde Colet. (2022) 27:2337–48. doi: 10.1590/1413-81232022276.14292021 35649021

[5] Costa GD Cotta RMM Reis JR Siqueira-Batista R Gomes AP Franceschini SCC. Avaliação do cuidado à saúde da gestante no contexto do Programa Saúde da Família. Ciênc Saúde Colet. (2009) 14:1347–57. doi: 10.1590/s1413-81232009000800007 19750343

[6] Ministério da Saúde. Assistência pré-natal. Divisão Nacional de Saúde Materno-Infantil, Secretaria Nacional de Programas Especiais de Saúde. Brasília, Brazil: Ministério da Saúde (1988).

[7] Ministério da Saúde. (2022) Nova versão da Caderneta da Gestante traz orientações sobre alimentação, saúde bucal, trabalho de parto e nascimento. Available from: https://www.gov.br/pt-br/noticias/saude-e-vigilancia-sanitaria/2022/05/nova-versao-da-caderneta-da-gestante-traz-orientacoes-sobre-alimentacao-saude-bucal-trabalho-de-parto-e-nascimento. [Accessed February 17, 2025]

[8] Bittencourt SDA Cunha EM Domingues RMSM Dias BAS Dias MAB Torres JA. Nascer no Brasil: continuity of care during pregnancy and postpartum period for women and newborns. Rev Saude Publica. (2020) 54:100. doi: 10.11606/s1518-8787.2020054002021, PMID: 33146323 PMC7584409

[9] Barreto FDFP Albuquerque RM. Discrepâncias entre o informe verbal e os registros no cartão da gestante, um instrumento negligenciado. Rev Bras Ginecol Obstet. (2012) 34:259–67. doi: 10.1590/S0100-72032012000600004 22801600

[10] Silveira MM Conrad NL Leite FPL. Effect of COVID-19 on vaccination coverage in Brazil. J Med Microbiol. (2021) 70:1466. doi: 10.1099/jmm.0.001466, PMID: 34845982

[11] Zajacova A Lawrence EM. The relationship between education and health: reducing disparities through a contextual approach. Annu Rev Public Health. (2018) 39:273–89. doi: 10.1146/annurev-publhealth-031816-044628, PMID: 29328865 PMC5880718

[12] Raghupathi V Raghupathi W. The influence of education on health: an empirical assessment of OECD countries for the period 1995–2015. Arch Public Health. (2020) 78:20. doi: 10.1186/s13690-020-00402-5, PMID: 32280462 PMC7133023

[13] World Health Organization. Global vaccine action plan 2030. Geneva, Switzerland: World Health Organization (2023).

[14] Costantino C Mazzucco W Bonaccorso N Cimino L Conforto A Sciortino M. Educational interventions on pregnancy vaccinations during childbirth classes improves vaccine coverages among pregnant women in Palermo’s province. Vaccines. (2021) 9:1455. doi: 10.3390/vaccines9121455, PMID: 34960202 PMC8707644

[15] Domingues CMAS Maranhão AGK Teixeira AM Fantinato FFS Domingues RAS. The Brazilian National Immunization Program: 46 years of achievements and challenges. Cad Saude Publica. (2020) 36:e00222919. doi: 10.1590/0102-311X00222919, PMID: 33111749

[16] Ministério da Saúde. (2025) Cobertura Vacinal – Residência. Available online at: http://tabnet.datasus.gov.br/cgi/dhdat.exe?bd_pni/cpnibr.def and https://infoms.saude.gov.br/extensions/SEIDIGI_DEMAS_VACINACAO_CALENDARIO_NACIONAL_COBERTURA_RESIDENCIA/SEIDIGI_DEMAS_VACINACAO_CALENDARIO_NACIONAL_COBERTURA_RESIDENCIA.html. [Accessed February 17, 2025].

[17] Boton Pereira DH Primo LS Pelizari G Flores E de Moraes-Vasconcelos D Condino-Neto A. Primary immunodeficiencies in a mesoregion of São Paulo, Brazil: epidemiologic, clinical, and geospatial approach. Front Immunol. (2020) 11:862. doi: 10.3389/fimmu.2020.00862, PMID: 32477349 PMC7235164

[18] Instituto Brasileiro de Geografia e Estatística. (2025). Portal do IBGE. Cidades e Estados/Portal de Mapas. Available online at: https://www.ibge.gov.br/cidades-e-estados/sp/.html? [Accessed January 19, 2025].

[19] Strassberg ER Power M Schulkin J Stark LM Mackeen AD Murtough KL. Patient attitudes toward influenza and tetanus, diphtheria, and acellular pertussis vaccination in pregnancy. Vaccine. (2018) 36:4548–54. doi: 10.1016/j.vaccine.2018.05.121, PMID: 29907484

[20] Karafillakis E Francis MR Paterson P Larson HJ. Trust, emotions, and risks: pregnant women’s perceptions, confidence, and decision-making practices around maternal vaccination in France. Vaccine. (2021) 39:4117–25. doi: 10.1016/j.vaccine.2021.05.096, PMID: 34099326

[21] Fundação Oswaldo Cruz (FIOCRUZ). (2025). Perguntas e respostas sobre vacinação. Available from: https://portal.fiocruz.br/perguntas-e-respostas-sobre-vacinacao?page=2. [Accessed February 03, 2025].

[22] Rocha BC Carvalheira AP Ferrari AP Tonete VL Duarte MT Parada CM. Cobertura vacinal e fatores associados em puérperas de município Paulista [immunization coverage and associated factors of women who have recently given birth in a city in São Paulo state]. Ciênc Saúde Colet. (2016) 21:2287–92. doi: 10.1590/1413-81232015217.16862015 27383361

[23] Santos Neto ET Oliveira AE Zandonade E Gama SGN Leal MC. O que os cartões de pré-natal das gestantes revelam sobre a assistência nos serviços do SUS da Região Metropolitana da Grande Vitória, Espírito Santo, Brasil? Cad Saude Publica. (2012) 28:1650–62. doi: 10.1590/s0102-311x2012000900005 23033181

[24] Guzman-Holst A DeAntonio R Prado-Cohrs D Juliao P. Barriers to vaccination in Latin America: a systematic literature review. Vaccine. (2020) 38:470–81. doi: 10.1016/j.vaccine.2019.10.088, PMID: 31767469

[25] Razzaghi H Kahn KE Calhoun K Garacci E Skoff TH Ellington SR. Influenza, Tdap, and COVID-19 vaccination coverage and hesitancy among pregnant women—United States, April 2023. MMWR Morb Mortal Wkly Rep. (2023) 72:1065–71. doi: 10.15585/mmwr.mm7239a4, PMID: 37768879 PMC10545430

[26] Seravalli V Romualdi I Ammar O De Blasi C Boccalini S Bechini A. Vaccination coverage during pregnancy and factors associated with refusal of recommended vaccinations: an Italian cross-sectional study. Vaccine X. (2024) 18:100483. doi: 10.1016/j.jvacx.2024.100483, PMID: 38623567 PMC11016930

[27] Tsiaousi I Psarris A Theodora M Antsaklis P Sindos M Koutroumanis P. COVID-19 vaccination acceptance during pregnancy in Europe. Cureus. (2024) 16:e63562. doi: 10.7759/cureus.63562, PMID: 39087190 PMC11289694

[28] Borges MASB Florentino PTV Cerqueira-Silva T Carvalho LF Araújo Oliveira V Aguilar GMO. Factors associated with COVID-19 vaccination among pregnant women in Rio de Janeiro City, Brazil. Sci Rep. (2023) 13:18235. doi: 10.1038/s41598-023-44370-6, PMID: 37880238 PMC10600223

[29] Fernandes FCGM Santos EG Barbosa IR. Age of first pregnancy in Brazil: data from the national health survey. J Hum Growth Dev. (2019) 29:304–12. doi: 10.7322/jhgd.v29.9523

[30] Soares Santana R Briguenti Souza K Lussari F Fonseca ES Andrade CO Meidas MMK. Cases and distribution of visceral leishmaniasis in western São Paulo: a neglected disease in this region of Brazil. PLoS Negl Trop Dis. (2021) 15:e0009411. doi: 10.1371/journal.pntd.0009411, PMID: 34129604 PMC8232419

[31] Jacob LMDS Santos AP Lopes MHBM Shimo AKK. Socioeconomic, demographic and obstetric profile of pregnant women with hypertensive syndrome in a public maternity. Rev Gaucha Enferm. (2020) 41:e20190180. doi: 10.1590/1983-1447.2020.20190180, PMID: 32491150

[32] Fauzia Malik A Belizan M Gutierrez M Vilajeliu A Sanclemente LN Gonzalez Casanova I. Pregnant women’s perspectives about maternal immunization in Latin America. Vaccine. (2021) 39:B44–9. doi: 10.1016/j.vaccine.2020.09.009, PMID: 32972734

[33] Silva TPRD Dumont-Pena E Moreira AD Camargos BA Meireles MQ Souza KV. Factors associated with normal and cesarean delivery in public and private maternity hospitals: a cross-sectional study. Rev Bras Enferm. (2020) 73:e20180996. doi: 10.1590/0034-7167-2018-0996, PMID: 32756742

[34] Vilajeliu A Magariños M Jauregui B Guzmán L Whittembury A Cain E. Enablers and barriers of maternal and neonatal immunization programs in Latin America. Vaccine. (2021) 39:B34–43. doi: 10.1016/j.vaccine.2020.07.051 32943263

